# The Premature Infants’ Gut Microbiota Assembly and Neurodevelopment (PIGMAN) Cohort Study: Protocol for a Prospective, Longitudinal Cohort Study

**DOI:** 10.3390/children12121644

**Published:** 2025-12-03

**Authors:** Tingting Li, Liangfeng Fang, Xianhong Chen, Youming He, Xiaoyuan Pang, Ling Lin, Heng Chen, Yajie Su, Yan Huang, Yanping Guo, Tiantian Xiao, Aiping Liu, Yanli Wang, Hanhua Yang, Chuan Nie, Wei Zhou, Guang Yang, Chunquan Cai, Xiaoguang Zhou, Shujuan Zeng, Yongfu Yu, Long Li, Huifeng Zhang, Lijun Yu, Guoqiang Cheng, Wenhao Zhou, Cheng Chen, Zhangbin Yu, Mingbang Wang, Yingmei Xie

**Affiliations:** 1 Longgang District Maternity & Child Healthcare Hospital of Shenzhen City (Affiliated Shenzhen Women and Children’s Hospital (Longgang) of Shantou University Medical College), Shenzhen 518172, China; litingting@lg.gov.cn (T.L.); fangliangfeng@lg.gov.cn (L.F.);; 2 Shenzhen Clinical Medical College, Guangzhou University of Chinese Medicine, Shenzhen 518116, China; 3 Division of Neonatology, Longgang Central Hospital of Shenzhen, Shenzhen 518116, China; 4 Department of Neonatology, Children’s Hospital of Xinjiang Uygur Autonomous Region (Xinjiang Hospital of Beijing Children’s Hospital), Urumqi 830054, China; yajiesu922@163.com (Y.S.);; 5 Shenzhen Baoan Women’s and Children’s Hospital, Shenzhen 518100, China; 6 Peking University Shenzhen Hospital, Shenzhen 518000, China; 7 Department of Neonatology, Chengdu Women’s and Children’s Central Hospital, School of Medicine, University of Electronic Science and Technology of China, Chengdu 611731, China; xiaott@uestc.edu.cn; 8 Public Health Service Center, Shenzhen 518101, China; 9 Department of Pediatrics, South China Hospital, Medical School, Shenzhen University, Shenzhen 518111, China; yanliwang@szu.edu.cn; 10 Department of Neonatology, Pingshan District Maternal and Child Health Care Hospital of Shenzhen, Shenzhen 518122, China; 11 National Key Clinical Specialty Construction Project/Neonatology Department, Guangdong Women and Children Hospital, Guangzhou 511442, China; 12 Guangdong Neonatal ICU Medical Quality Control Center, Guangzhou 511442, China; 13 Department of Neonatology, Guangzhou Women and Children’s Medical Center, Guangzhou Medical University, Guangzhou 510623, China; 14 Department of Pediatrics, The First Medical Center of Chinese PLA General Hospital, Beijing 100853, China; yangguang74@301hospital.com.cn; 15 Senior Department of Pediatrics, The Seventh Medical Center of Chinese PLA General Hospital, Beijing 100700, China; 16 Medical School of Chinese People’s Liberation Army, Beijing 100853, China; 17 Tianjin Key Laboratory of Birth Defects for Prevention and Treatment, Tianjin Pediatric Research Institute, Tianjin Children’s Hospital (Children’s Hospital of Tianjin University), Tianjin 300134, China; ccq_6313@tju.edu.cn; 18 Department of Neonatology, The Eighth Affiliated Hospital, Sun Yat-sen University, Shenzhen 518033, China; 19 School of Public Health, Fudan University, 220 Handan Road, Shanghai 200433, China; yu@fudan.edu.cn; 20 Department of Pediatrics, The Second Hospital of Hebei Medical University, Shijiazhuang 230601, China; 21 Department of Pediatrics, Fourth Affiliated Hospital, Harbin Medical University, Harbin 150001, China; yulijunccd@163.com; 22 Department of Neonatology, Children’s Hospital of Fudan University, Shanghai 201102, China; 23 Department of Neonatology, Shenzhen People’s Hospital (The Second Clinical Medical College, Jinan University; The First Affiliated Hospital, Southern University of Science and Technology), Shenzhen 518000, China

**Keywords:** premature, neurodevelopmental disorders, brain-gut axis, prospective birth cohort, causal inference

## Abstract

Background: Early-life gut microbiota colonization plays a significant role in the neurodevelopment of infants and young children. However, the causal relationship between early-life gut microbiota colonization and neurodevelopment in preterm infants has not yet been conclusively established. Our research will initiate the PIGMAN (Premature Infants Gut Microbiota Assembly and Neurodevelopment) cohort study to systematically examine the dynamic interplay between gut microbiota developmental trajectories and neurodevelopmental processes in preterm infants. Methods: This study will employ a longitudinal cohort design and utilize data from the PIGMAN cohort, examining the interplay between gut microbiota metabolism and neurodevelopmental outcomes. The study design incorporates longitudinal stool sample collection, which will be analyzed through 16S rRNA gene sequencing and metagenomic shotgun sequencing, enabling comprehensive characterization of microbial community dynamics and functional metabolic pathways. Anticipated Results: Advanced analytical approaches incorporating causal inference methodologies will be implemented to identify significant microbial and metabolic biomarkers associated with neurodevelopmental outcomes in preterm neonates, and to establish causal pathways between these biomarkers and neurodevelopment. These analytical advancements will facilitate the construction of predictive models that utilize temporal microbial signatures and metabolite trajectories as prognostic indicators for neurodevelopmental outcomes. Causal inference method evaluations will further reveal that specific gut-derived metabolites, particularly those involved in cholesterol metabolism and neural signaling pathways—such as bile acids and GABA (gamma-aminobutyric acid)—exhibit superior predictive capacity for cognitive development trajectories. Anticipated Conclusions: The findings will collectively suggest that longitudinal metabolic profiling of the gut ecosystem, when combined with causal network analysis, provides a novel paradigm for developing clinically actionable predictive models of neurodevelopment in vulnerable preterm populations.

## 1. Introduction

The primordial assembly of the gut microbiome during early life serves as a fundamental determinant of infant developmental programming and lifelong health trajectories [1,2]. During the initial stages, the infant gut microbiota is characterized by limited species diversity and rapid compositional changes, progressing through distinct developmental phases before achieving a stable and diverse microbial ecosystem [3,4,5]. Crucially, this dynamic ecological succession operates within developmentally sensitive windows, particularly the primordial colonization phase (0–28 days postnatal) and microbial maturation phase (29 days to 24 months), wherein perturbations of microbial–host dynamics can induce epigenetic reprogramming with multiorgan consequences [6].

Global estimates for 2020 indicated 13.4 million preterm births and 23.4 million small-for-gestational-age neonates worldwide, with stagnant prevalence trends over the past decade [7]. There exist parallel early developmental windows for the gut microbiota and the nervous system during prenatal to postnatal of life. The degree of cerebral immaturity at birth critically determines neurodevelopmental trajectories in preterm infants [8]. Preterm-born individuals exhibit heightened susceptibility to atypical neurodevelopment [9], with emerging evidence implicating gut microbiome dysbiosis in modulating neural functions through microbiota–gut–brain axis interactions [10]. Such microbial perturbations may predispose neonates to long-term neurological sequelae, including sensory processing deficits and cognitive impairments [10]. Longitudinal studies in extremely low gestational age neonates have demonstrated that early neurodevelopmental alterations persist through childhood, manifesting as visual/auditory processing deficiencies and compromised learning capacities [11]. Therapeutic interventions targeting synchronized microbiome–brain developmental windows could offer a promising approach to mitigating neurodevelopmental risks in preterm infants.

The past three decades have witnessed a paradigm-shifting convergence between microbiology and neuroscience, catalyzed by groundbreaking discoveries in microbiota–gut–brain axis mechanisms [12]. Preterm infants represent a unique population in which optimization of initial colonization and microbiota development can affect brain development and enhance neurological outcomes [13]. Survivors of prematurity often encounter a lifetime of neurologic impairment [14]. While longitudinal birth cohort studies employing metagenomic sequencing have mapped characteristic gut microbiota succession trajectories in preterm infants [15,16,17], much remains unknown about the mechanism of the gut bacteriomes and neurodevelopment in infants.

Initiated in 2024, the PIGMAN Cohort Study represents a pioneering prospective longitudinal investigation designed to elucidate dynamic interactions between gut microbiome maturation and neurodevelopmental trajectories in preterm infants. This multidimensional study employs weekly longitudinal profiling of stool microbiomes during the critical first month of early life. Our study integrates 16S rRNA and shotgun metagenomic sequencing for microbial community characterization, metabolomic profiling for functional pathway analysis, and serial neurobehavioral assessments at 1, 3, and 6 months of postnatal age.

The protocol strategically incorporates seven temporal sampling points, employing causal inference to enhance understanding of the interactions between gut microbiota and the underlying developmental processes in premature growth and neurodevelopment. Through this longitudinal design, the study aims to identify predictive biomarkers linking early dysbiosis patterns to later neurocognitive outcomes.

## 2. Materials and Methods

### 2.1. Study Design

**Figure 1:**
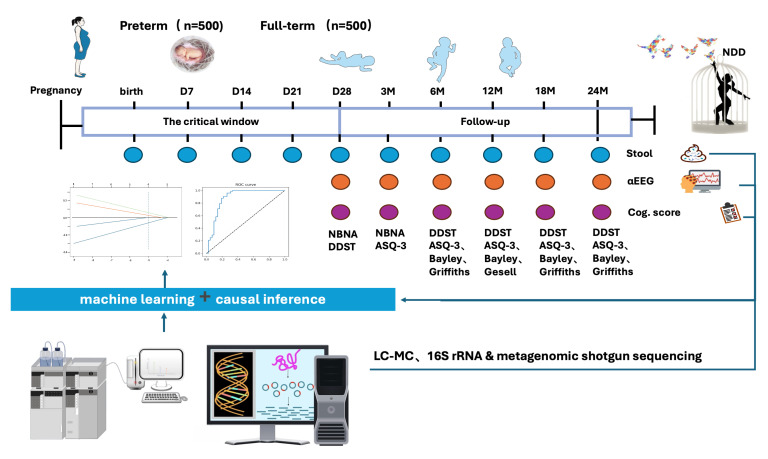
The flowchart of the PIGMAN Cohort Study design. The flowchart illustrates the design of the PIGMAN Cohort Study, which investigates the relationship between preterm infant gut microbiota and neurodevelopment within the ‘microbiome–gut–brain’ axis framework. The study enrolls 500 preterm and 500 full-term infants, with longitudinal sampling of stool for multi-omics analysis, combined with neurobehavioral assessments at multiple time points from birth to 24 months. Machine learning and causal inference approaches will be applied to identify key microbial and metabolic factors linked to neurodevelopmental outcomes. Abbreviation: NBNA, Neonatal Behavioral Neurological Assessment; DDST, Denver Developmental Screening Test; ASQ-3, Ages and Stages Questionnaires, Third Edition; Griffiths, Griffiths Mental Development Scales; Bayley, Bayley Scales of Infant and Toddler Development; Gesell, Gesell Developmental Schedules; αEEG, α-Electroencephalogram; Cog. Score, Cognitive Score; NDD, Neurodevelopmental Disorder; LC-MC, Liquid Chromatography–Mass Spectrometry.

In this study, participants will be recruited from July 2025 to September 2028. Stool samples from premature infants will be collected at ten scheduled timepoints (days 0, 7, 14, 21, 28; month 3, 6, 12, 18, 24). Neurodevelopmental assessments will be conducted using αEEG monitoring and standardized scales—including the Neonatal Behavioral Neurological Assessment (NABA), the Denver Development Screen Test (DDST), and the Infant Heart Scale—at months 3, 6, 12, 18, and 24. We will leverage advanced artificial intelligence techniques to systematically analyze multi-omics data from our prospective cohort. Furthermore, these results will be integrated with previously accumulated and published multi-omics data on the “microbiome–brain–gut” axis to gain deeper insights into the relationship between preterm microbiota and neurodevelopmental trajectories (Figure 1 illustrates the study design flowchart).

### 2.2. Participants

In prospective birth cohort studies of preterm birth and neurodevelopmental outcomes, we meticulously adhere to scientific ethics when recruiting participants. The inclusion criteria require that infants have no pre-existing high-risk factors, no history of antibiotics exposure, and no occurrence of fetal defecation prior to enrollment. Eligible participants comprise very premature infants born between 24 weeks’ and 31^+6^ weeks’ gestation, with birth weights ranging from 500 g and 1500 g. Detailed data on infant feeding type (categorized as exclusive breastfeeding, exclusive formula-feeding, or mixed feeding) are prospectively collected at all timepoints to account for its recognized critical role in gut microbiota development. A homogeneous cohort is established to enable the systematic collection of fecal samples at five standardized timepoints (days 0, 7, 14, 21, and 28) during the first postnatal month.

Subjects will be excluded based on the following criteria: (1) congenital heart disease requiring neonatal surgical intervention (e.g., hemodynamically significant PDA or complex cyanotic defects), while asymptomatic infants with minor defects will be included; (2) confirmed genetic disorders including trisomy 21, other major chromosomal abnormalities (e.g., trisomy 18/13), or growth-affecting syndromes (e.g., Russell–Silver syndrome), as these inherently alter neurodevelopment; (3) severe neurological injury predictive of cerebral palsy, since CP cannot be diagnosed neonatally; (4) moderate-to-severe perinatal asphyxia, defined as Apgar ≤ 3 at 5 min or requirement for therapeutic hypothermia, due to distinct developmental risks; or (5) structural anomalies impacting feeding. Mothers using tobacco/alcohol >1/week during pregnancy or breastfeeding will also be excluded. These criteria will be applied at enrollment to minimize confounding effects on growth and microbiota development, ensuring the study focuses on prematurity-related outcomes. For premature infants, we will take extra precautions, including developing a comprehensive medical plan to safeguard the health and well-being of our study subjects throughout the research period.

### 2.3. Biological Samples Collection and Management

Stool samples from preterm infants will be collected by researchers at specified timepoints: days 0, 7, 14, 21, and 28, as well as at 3 and 6 months after birth. All collections will be conducted between 6:00 AM and 6:00 PM. Using standard fecal collectors, midstream stool samples will be obtained from spontaneously passed stools, with a target weight of 5 g; however, a minimum of 0.5 g will be accepted when 5 g is not feasible, and the actual weight of each sample will be recorded. Following collection, samples will be stored at −80 °C within 30 min for long-term preservation.

For post-discharge sampling at the 3- and 6-month timepoints, the following procedure will be implemented: Specialized research staff will conduct scheduled phone calls with parents to coordinate sample collection. Standardized fecal collection kits, including detailed pictorial and written instructions, sterile collectors, and DNA/RNA preservation solution, will be mailed to families. Parents will be instructed to collect midstream stool samples following the same protocol as used in the hospital, place the sample immediately into the provided preservative, and record the collection time.

All community-collected samples will be returned to the study center via temperature-monitored express cold chain delivery within 24 h of collection. Upon receipt, samples will be logged into the laboratory information management system, aliquoted, and stored at −80 °C before batch shipment on dry ice to the designated sequencing provider for 16S rRNA gene sequencing and shotgun metagenomic analysis.

Detailed documentation will be maintained for all biological samples, including specific collection and storage times, recorded both on sample containers and in corresponding registration documents. Furthermore, comprehensive clinical data will be prospectively collected, including infant vaccination status and all documented infection episodes, as these factors are recognized as important covariates influencing both microbiome composition and neurodevelopmental outcomes. Prior to enrollment, informed consent will be obtained from the infant’s caregivers. Upon obtaining consent, biological samples will be promptly collected following standardized protocols.

### 2.4. Data Collection

The primary outcomes will include neurodevelopmental abnormalities, amplitude-integrated EEG (aEEG) monitoring, and standardized neurodevelopmental scales. Microbiome development patterns will be assessed through 16S rRNA sequencing of serial fecal samples (Days 0, 7, 14, 21, 28) to characterize microbial succession dynamics, measuring alpha/beta diversity and taxonomic composition. Early neurological function will be assessed via aEEG at 39–41 weeks postmenstrual age. The evaluation will include sleep–wake cycling maturity, background pattern classification, and seizure activity, and all analyses will be performed by certified neonatal neurologists.

These outcomes are carefully selected to provide both short-term neurological assessment and long-term developmental evaluation using validated, clinically meaningful measures. Neurodevelopmental status at 24 months postnatal age will be assessed using the Bayley Scales of Infant and Toddler Development, Third Edition (BSID-III) to evaluate cognitive, language, and motor development (composite scores) at 24 ± 1 months by certified examiners blinded to microbiome data. All enrolled infants will undergo aEEG evaluation at 28 days postnatal age unless contraindicated (e.g., by scalp edema or parental refusal), with continuous 4 h recordings during quiet sleep states to assess waveform continuity, sleep–wake cycle maturity, and voltage amplitude. Neurodevelopmental outcomes will be further evaluated using the Bayley Scales of Infant and Toddler Development (BSID-III) and Gesell Developmental Schedules (GDS) at 6, 12, and 24 months’ postnatal age, providing comprehensive longitudinal assessment of cognitive, language, motor, and social–emotional development.

Secondary outcomes will include the following: (1) Growth parameters: Weight, length, and head circumference z-scores will be measured monthly until 6 months, then at 12 and 24 months, to evaluate postnatal growth patterns. (2) Severe complications: The incidence of necrotizing enterocolitis (NEC), retinopathy of prematurity (ROP), intracranial hemorrhage (IVH) and birth asphyxia will be documented during NICU stay and at discharge, to assess their neurodevelopmental impact. (3) Maternal/infant characteristics: Demographics, pregnancy history, delivery mode, and antibiotic exposure will be collected at enrollment to identify potential confounders. Baseline data will include maternal age, education, medication history, delivery mode, gestational age, birth measurements, Apgar scores, and postnatal complications.

Trained researchers will conduct an aEEG interpretation (classified as abnormal if discontinuous waveforms, immature sleep cycles, or reduced amplitude [≤10 μV upper margin/≤5 μV lower margin] are observed) and administer neurodevelopmental assessments at scheduled follow-ups. These measures will ensure comprehensive evaluation of prematurity-related outcomes while controlling for confounding factors.

### 2.5. Sample Size Estimation

This prospective longitudinal cohort study is designed to enroll an estimated sample size of 500 preterm infants to ensure robust detection of neurodevelopmental outcomes. The sample size calculation is based on an expected 25% prevalence of neurodevelopmental disorders in preterm infants [18], with parameters set to detect a 15% absolute difference between subgroups at 80% power (β = 0.20) and α = 0.05 significance (two-tailed). While the initial G*Power(v 3.1.9.7) analysis indicated a minimum requirement of 164 infants, we have increased enrollment to 500 to account for an anticipated 30% attrition over the 2-year follow-up period, enable comprehensive subgroup analyses by gestational age strata, and enhance precision for secondary outcomes with smaller effect sizes. The study will recruit infants from January 2025 to September 2028, with data analysis scheduled for completion by July 2029. To address multiple comparisons, we will maintain α = 0.05 for primary outcome analyses, apply Bonferroni correction for secondary outcomes based on the number of comparisons, and clearly label exploratory analyses as hypothesis-generating. This approach ensures adequate power for our primary neurodevelopmental assessment while allowing the examination of modifier effects and maintaining statistical rigor across all analyses.

### 2.6. Ethics Approval

The institutional review boards at Longgang District Maternity & Child Healthcare Hospital of Shenzhen City (Affiliated Shenzhen Women and Children’s Hospital [Longgang] of Shantou University Medical College) has approved all protocols for the Premature Infants Gut Microbiota Assembly and Neurodevelopment (PIGMAN) Cohort Study (Ethics Code: LGFYKYXMLL-2024-50). Written informed consent for participation in this study will be obtained from all participants. This study adheres to the Strengthening the Reporting of Observational Studies (STROBE) reporting guidelines for cohort studies.

### 2.7. Statistical Analysis

Statistical analyses will employ appropriate tests based on data distribution: continuous variables will be presented as mean (SD) or median (IQR), while categorical variables as frequency (%), using *t*-tests/Mann–Whitney U-tests or χ^2^/Fisher’s exact tests, respectively. Correlation analyses (Pearson/Spearman) will examine preterm birth–neurodevelopment associations, with multivariable regression adjusting for confounders, including infant vaccination status, infection episodes, and feeding patterns.

In microbiome analyses, 16S rRNA sequencing data will be processed with QIIME 2 (v2024.2) to evaluate alpha- and beta-diversity. Specifically, alpha diversity will be assessed using Chao1 richness index, Shannon diversity index, and Faith’s Phylogenetic Diversity, while beta diversity will be evaluated through Bray–Curtis dissimilarity and both weighted and unweighted UniFrac distances, with statistical significance tested via PERMANOVA. These analyses will enable the systematic comparison of microbial community structure and composition across different gestational age groups and sampling timepoints. Multivariate ordination, including Principal Coordinates Analysis (PCoA) and Non-Metric Multidimensional Scaling (NMDS), will be applied to visualize and test community-level differences. Infants will be stratified by gestational age (<28 weeks, 28–32 weeks, >32 weeks) and sex for specific analyses. Key microbial features—such as relative abundances of neurodevelopment-associated taxa (e.g., *Bifidobacterium*), enterotypes, and dysbiosis indices—will be linked to neurodevelopmental scores via adjusted regression models. To account for multiple comparisons in taxa-level association analyses, the false discovery rate (FDR) will be controlled using the Benjamini–Hochberg procedure, with an FDR-adjusted *p*-value (q-value) < 0.05 considered statistically significant.

Given the observational design, causal inference will be limited to mediation models evaluated using structural equation modeling (SEM) to explore the potential mediating role of microbiota in pathways linking prematurity and neurodevelopment. Sensitivity analyses will assess unmeasured confounding (E-values) and examine robustness to missing data and model specifications. Subgroup analyses will focus on gestational age strata, sex differences, and perinatal risks.

All analyses and visualizations will be performed in R version 4.4.2, utilizing the following key packages: ggplot2 (v3.3.5), labdsv (v2.0), ape (v5.5), MaAsLin2 (v1.7.3), tidyverse (v1.3.1), SIAMCAT (v2.0), randomForest (v4.7), glmmLasso (v1.6), and lavaan (v0.6).

## 3. Anticipated Results

### 3.1. Neurodevelopmental Risks and Gut–Brain Axis Mechanisms in Preterm Infants

Compared to term infants, premature infants face a higher risk of both short-term and long-term adverse neurodevelopmental outcomes [19,20,21]. Premature infants, including late-preterm infants, are susceptible to various conditions, including brain injury, and exhibit a high incidence of neurodevelopmental maldevelopment [9,22]. Neurodevelopmental disorders represent common long-term neurological complications in premature infants, encompassing intellectual developmental disorders [23], developmental speech or language disorders [24], autism spectrum disorder [25], developmental learning disorders [9,26], attention-deficit/hyperactivity disorder [27], tic disorders, and other neurodevelopmental disorders [28].

A growing body of evidence suggests that early gut microbiota acquisition and development occur synchronously with neurodevelopmental processes. Gut-derived microbial metabolites can indirectly influence the brain by stimulating the enteric nervous and immune systems [29,30]. For instance, the abundance of *E. coli* and *Klebsiella* has been correlated with deep gray matter and cortical microstructural parameters in preterm infants, possibly influencing brain development through metabolic pathways [31]. Additionally, gut microbes produce neuroactive substances such as acetylcholine and serotonin, which may directly or indirectly modulate brain function [32].

### 3.2. Longitudinal Microbial Dynamics in Early Development

While existing studies have demonstrated temporal associations between gut microbiota composition and neurodevelopmental outcomes at various intervals—including 1 month [33], 3 months [17], 6 months [34], and 12 months [35]—important questions remain unanswered regarding how gestational age specifically shapes microbial colonization patterns throughout the crucial early developmental period from birth to 3.5 years of age [33].

To address this gap, our study employs an innovative longitudinal design with systematic fecal sampling at five neonatal timepoints (days 0, 7, 14, 21, and 28 postpartum). This approach enables characterizing the dynamic progression of the gut microbiota in preterm infants and facilitates elucidating its relationship with neurological development. Beyond confirming previously reported correlations, we incorporate advanced analytical methodologies that have demonstrated promise in overcoming the limitations of traditional diagnostic approaches [36] and in decoding complex microbiome–host interactions.

### 3.3. Microbial Mechanisms and Neurodevelopmental Prediction

We anticipate that our advanced analytical methodologies—including machine learning approaches and multivariate ordination techniques—will enable multidimensional analysis of microbial community dynamics, potentially revealing novel biomarkers for neurodevelopmental trajectories. By integrating longitudinal microbial profiling with neurodevelopmental assessments, this study aims to not only to map the temporal evolution of gut–brain axis interactions in preterm infants but also to identify mechanistic pathways underlying microbiota-mediated neurological outcomes—thereby bridging the current gap between correlative observations and actionable clinical insights.

Notably, although preterm and term infants exhibit generally comparable gut microbiota developmental trajectories during early postnatal months, preterm neonates demonstrate significantly reduced microbial diversity. This ecological simplification primarily results from prolonged neonatal intensive care unit stays and exposure to potent microbiota-modifying factors—including gestational immaturity, antibiotic overexposure, and suboptimal feeding practices.

These clinical realities underscore the necessity for developing stratified microbial–neurodevelopmental prediction models that account for critical confounding variables, such as infant vaccination status, infection episodes, and feeding patterns. Future investigations should prioritize the integration of longitudinal cohort designs with multi-omics approaches combined with immune marker profiling and bidirectional modeling of microbiome–immune relationships to identify novel therapeutic targets. Promising avenues include the development of microbial metabolite-based biomarkers for early neuroprognostication, and the exploration of precision nutritional strategies targeting microbial modulation. Such microbiota-directed interventions may ultimately enhance neurodevelopmental outcomes in this vulnerable population through optimized gut–brain axis programming during critical developmental windows.

## 4. Anticipated Problems

### 4.1. Limitations of Neurodevelopmental Assessment

While conventional neurodevelopmental scales provide valuable initial phenotypic assessments, their application in preterm populations is limited by reduced sensitivity in detecting graded neurological maturation patterns and their inability to quantitatively characterize microbial–neural interactions. To overcome these limitations, we have incorporated high-temporal-resolution EEG monitoring to capture subtle neurophysiological fluctuations during spontaneous activity cycles, enabling more precise mechanistic investigation of gut–brain axis signaling through integrated analysis of microbial diversity metrics and neural oscillatory biomarkers.

However, this enhanced approach introduces significant operational complexities, including the substantial financial costs of continuous EEG acquisition and the analytical challenges associated with processing terabyte-scale electrophysiological datasets, which we plan to address through optimized recording protocols and cloud-based parallel processing.

### 4.2. Challenges in Post-Discharge Sampling

The longitudinal fecal sampling protocol—spanning days 0, 7, 14, 21, 28, as well as 3 and 6 months post-discharge—provides critical temporal resolution but presents unique challenges in maintaining specimen integrity. Particular concerns arise during post-discharge collection periods, where variable parental compliance in community settings may lead to increased sample attrition, and deviations from standardized cryopreservation protocols during home-based storage (>20 °C temperature variations) may compromise microbial viability and alter composition.

These factors may introduce non-random missing data patterns and storage-related artifacts that must be carefully considered when interpreting longitudinal microbial dynamics. To mitigate these risks, we will provide participants with standardized collection kits equipped with temperature loggers, implement a comprehensive sample tracking system with regular parental reminders, and apply stringent quality control measures including the exclusion of samples showing evidence of significant temperature deviation.

### 4.3. Participant Retention and Data Missingness

Ensuring complete follow-up after hospital discharge represents another critical challenge that could impact study outcomes. We will employ multiple strategies to maximize retention, including scheduled community health worker visits, telehealth consultations, and systematic comparison of characteristics between study completers and those lost to follow-up to assess potential bias.

For handling missing outcome data, we will use appropriate multiple imputation methods, where justified, and conduct sensitivity analyses to evaluate the robustness of our findings. These comprehensive measures are designed to maintain methodological rigor while addressing the practical challenges inherent in longitudinal investigations of preterm infant development and gut microbiota dynamics.

## 5. Conclusions

Despite collective efforts to improve both maternal and postnatal care, preterm birth rates and the associated neurodevelopmental disabilities continue to rise. The gut–brain connection plays a particularly important role in maintaining brain health, with strong evidence linking gut dysbiosis to brain disorders. Longitudinal profiling of gut microbial ecosystems in preterm neonates enables predictive modeling of neurodevelopmental trajectories, facilitating early implementation of microbiome-targeted interventions to mitigate adverse neurodevelopmental sequelae associated with aberrant cortical maturation. This predictive framework capitalizes on the dynamic interplay between microbial metabolites and neurodevelopmental outcome, allowing risk stratification during critical neuroplasticity windows. Based on the research findings, we will characterize the growth dynamics of gut microbiota in preterm infants during the first postnatal month. Through precise modulation of microbial communities via probiotic supplementation and human milk oligosaccharide interventions, we will establish a novel therapeutic paradigm to recalibrate gut–brain axis signaling pathways, thereby promoting normative neural circuit formation and reducing the incidence of white matter injury.

## Data Availability

The original contributions presented in this study are included in the article/Supplementary Materials. Further inquiries can be directed to the corresponding authors.

## Supplementary Materials

The following supporting information can be downloaded at: https://www.mdpi.com/article/10.3390/children12121644/s1; File S1: Fecal sample collection process.
