# Supplementary material for: The Premature Infants’ Gut Microbiota Assembly and Neurodevelopment (PIGMAN) Cohort Study: Protocol for a Prospective, Longitudinal Cohort Study
Source: Children (Basel). 2025 Dec 3;12(12):1644. doi: 10.3390/children12121644 (PMC12732262; doi:10.3390/children12121644)
Supplement: Supplementary file 1 [file children-12-01644-s001.zip › children-3845722-supplementary.pdf]

Supplementary File S1

Fecal sample collection process

I. Important Notes

1. Upon receipt of the sample collection tubes (anaerobic preservation fluid tube and regular tubes), **immediately remove the anaerobic sterile preservation fluid tube and store it in the freezer compartment** of a refrigerator. The regular sampling tubes can be stored at room temperature. **Do not discard the foam box**, as it will be used for subsequent sample return.
2. **Collect mid-stream stool samples once into three tubes** (1 anaerobic preservation fluid tube and 2 disposable sterile regular tubes). For each tube, collect **at least one scoop of stool**; it is preferable to collect more while avoiding contamination. **Label the tubes clearly and write the sampling date**.
   a) **Note for the anaerobic sterile tube:** After collecting the sample, **close the tube cap securely and shake it vigorously (up and down) until the sample breaks down into fine particles/flocs and mixes thoroughly with the preservation fluid. Do not discard the bag and desiccant** that came with the anaerobic tube; place the sampled tube back into them after collection.
   b) **Note for the regular tubes:** No additional processing is required after sample collection.
3. **After collection, all samples must be stored frozen** (in the freezer compartment of a household refrigerator, set to the maximum setting, approximately -20°C). They can be stored for up to one week under these conditions.
4. **Sample Shipment After Collection:**

5. **Within Shenzhen:** Ship with ice packs. If ice packs are unavailable, the day before sample pickup, place several sealed bottles of water in the freezer compartment until completely frozen. During shipment, pack these frozen water bottles together with the samples in the packaging box/bag for insulation.

6. **Outside Shenzhen:** Ship with dry ice. After sample collection is complete, please contact a project team member via WeChat to place an order for dry ice. Do not ship the samples until the dry ice has arrived. Coordinate with the project team member to arrange the courier shipment.

II. Figure Legend

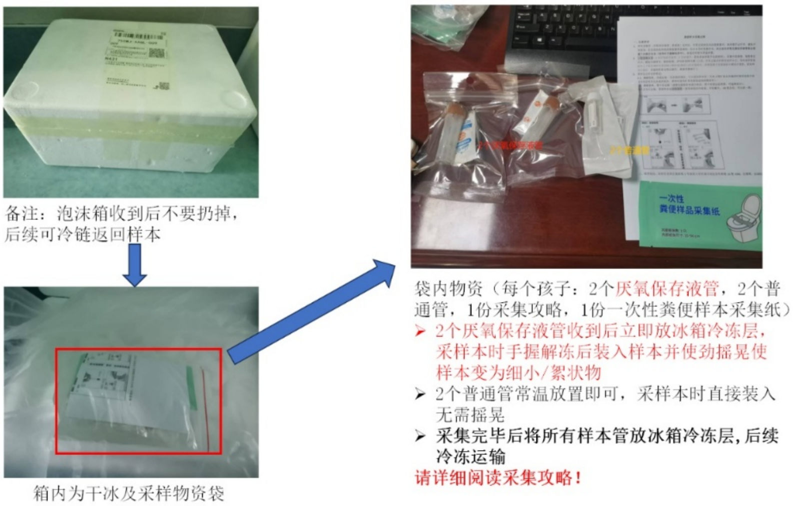

Figure S1. Stool sample collection kit and pre-collection instructions. The kit contains two anaerobic preservation fluid tubes (to be stored frozen upon receipt), two regular tubes (storable at room temperature), a detailed collection guide, and a disposable fecal collection paper. Critical handling notes are provided, emphasizing the need to retain the foam box for cold-chain sample return and to freeze all samples post-collection.

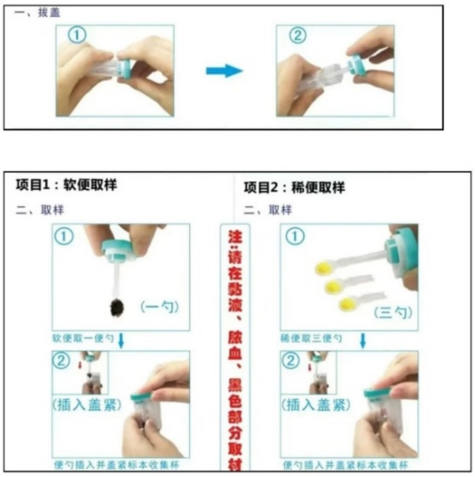

Figure S2. Detailed specimen collection protocol. The collection process involves using the integrated spoon to obtain the sample. For formed/soft stool, a single spoonful is sufficient. For liquid/loose stool, three spoonfuls are required. After sampling, the spoon is carefully inserted back into the specimen collection cup, and the cap is closed firmly to ensure a tight seal.
